# The safety and efficacy of spray cryotherapy after endoscopic sinus surgery in chronic rhinosinusitis: A systematic review of randomized controlled trials

**DOI:** 10.12688/f1000research.143321.1

**Published:** 2024-01-03

**Authors:** Mohammad J. J. Taha, Obaida Falah, Mohammad T. Abuawwad, Ayham R. Sara, Adham A. Aljariri, Abdulqadir J. Nashwan, Ibrahim T. Abuawwad, Ahmad J. Taha, Abdullah A. Elhakim, Majed Akili

**Affiliations:** 1 Department of Clinical Medicine, Kasr Alainy Faculty of Medicine, Cairo University, Giza, Giza Governorate, 112631, Egypt; 2 Otolaryngology Department, Hamad Medical Corporation, Doha, 3050, Qatar; 3 Nursing Department, Hazm Mebaireek General Hospital, Hamad Medical Corporation, Doha, 3050, Qatar

**Keywords:** chronic rhinosinusitis, functional endoscopic sinus surgery, middle meatus antrostomy, nasal polyposis, spray cryotherapy

## Abstract

**Background:**

Chronic rhinosinusitis (CRS) is a condition that affects 5–12% of the general population. Endoscopic sinus surgery (ESS) is the preferred treatment because of its few adverse effects. The most common post-operative consequences include synechia, nasal blockage, and disease recurrence. Spray cryotherapy is a novel therapeutic approach with promising outcomes for the treatment of upper airway disorders.

This review aimed to investigate the effects of spray cryotherapy (SCT) following ESS in patients with chronic rhinosinusitis.

**Methods:**

Six electronic databases were searched for randomized clinical trials (RCTs). The selected trials were evaluated for methodological quality, and data were extracted by two independent reviewers. The Cochrane risk-of-bias tool was used to assess the quality of evidence.

**Results:**

Three RCTs with 85 patients were included in the final analysis. SCT was related to -16 and -77 reductions in Lund-McKay and SNOT-22 scores after 36 weeks of follow-up, in contrast to a placebo, which showed -10.4, -65. Regarding the side effects of SCT, no adverse effects were reported, and visual assessments showed no pain, visual field loss, or any other ocular complications.

**Conclusions:**

SCT is a new treatment modality after endoscopic sinus surgery that shows an effective post-operative management strategy with better post-operative scales (Lund-McKay, SNOT-22, POSE, and Lund-Kennedy) and less edema, obstruction, crusting, and inflammation with minimal or no side effects. However, further research with longer follow-ups, a larger sample size, and subjective assessment is needed to assess any possible long-term side effects.

## Introduction

Chronic rhinosinusitis (CRS) is a name describing a family of clinical conditions that affect 5–12% of the general population, disturbing their quality of life and adding a financial burden to the healthcare system. ^1^ Chronic rhinosinusitis in adults was defined in the European position paper on rhinosinusitis and nasal polyps 2020 as “the presence of two or more symptoms, one of which should be either nasal blockage, obstruction, congestion, or nasal discharge (anterior or posterior nasal drip), with or without facial pain/pressure, and with or without a reduction or loss of smell; for 12 weeks; with validation by telephone or interview.”. ^1^ CRS can occur with or without polyps, and there appears to be a significant overlap between the two forms of chronic rhinosinusitis in terms of the inflammatory profile, clinical presentation, and treatment effect. Despite these differences in etiology and phenotype, many treatments for chronic rhinosinusitis are initiated in clinical practice without knowledge of a patient’s “polyp status,” despite the fact that 25–30% of CRS cases present with nasal polyps (NP). ^2^ CRS has many variations in terms of histology and clinical presentation, making its management controversial. Medically, many options are available, including corticosteroids, antibiotics, antihistamines, anti-leukotrienes, decongestants, saline, and aspirin, while surgical options include primary sinus surgery, revision endoscopic surgery, and many other techniques. ^1^^,^^3^ In the present study, the surgery of interest is endoscopic sinus surgery (ESS) for CRS. Despite being widely utilized with over 250,000 sinus surgeries per year in the US, ^4^ it is usually associated with synechia, obstruction, and stenosis of the maxillary or frontal ostium, which is caused by the apposition of two mucosal surfaces. Excessive scar formation, adhesions, and sinus osteomeatal stenosis are considered the main causes of disease recurrence and the need for revision surgery. ^5^

Spray cryotherapy (SCT) is a technique that involves treating mucosal lesions with liquid nitrogen at -90°C to freeze cellular water content and impose cellular necrosis for a few minutes to prevent mucosal surface adhesion, resulting in faster healing, less obstruction, and stenosis. ^6^ It was previously adapted for the treatment of esophageal lesions, including esophageal cancer and Barrett’s esophagus. ^7^^–^^9^ In 2010, SCT was first used by Krimsky *et al*. for the treatment of glottic and subglottic stenosis, and was the first application of SCT in airway surgery. ^10^ Subsequently, SCT was successfully used in cases of benign and malignant airway diseases. ^11^^,^^12^ In this systematic review of randomized controlled trials, we summarized and analyzed the available evidence regarding the impact of SCT after ESS for CRS with or without polyps. To our knowledge, this is the first systematic review to address the effects of SCT on healing after ESS surgery.

## Methods

### Study design

This systematic review of randomized controlled trials was performed in accordance with the PRISMA checklist. ^13^^,^^14^ The filtration phases were carried out according to the Cochrane criteria. ^15^

### Inclusion criteria

We included studies that examined the effectiveness of post-operative SCT in patients with chronic rhinosinusitis of either sex, regardless of age, from any healthcare context. The main outcome of interest was investigating the main impact of SCT after endoscopic sinus surgery for chronic rhinosinusitis with or without nasal polyposis. According to our inclusion criteria, the studies included satisfied the following criteria: 1) randomized control trials (RCT); 2) SCT was used following endoscopic sinus surgery; and 3) Only in English.

### Search strategy and study selection

The PubMed, Scopus, ClinicalTrails.gov, Cochrane Library, Web of Science, and Google Scholar databases were used for the searches, which had a date range from inception until 1/8/2023 for all databases. Randomized controlled trials, endoscopic sinus surgery (mesh terms), and spray cryotherapy (mesh terms) were used as search terms. For Google Scholar, Web of Science and ClinicalTrials.gov we used the simplest of keywords “spray cryotherapy and chronic rhinosinusitis” without filter. For PubMed and Scopus “(((spray cryotherapy) or (SCT)) and ((chronic rhinosinusitis) or (chronic rhinitis)) and ((endoscopic sinus surgery) or (endoscopic sinus surgery (MESH)))” were used. We also manually searched the entire text of the recognized systematic reviews that had been published in the area for potentially relevant details. Following the searches, references were located and exported to an Endnote X9 file after duplicates were eliminated. Filtration and extraction were performed by two independent reviewers, and any disagreement was resolved by a third reviewer.

### Quality assessment

The Cochrane Handbook for Systematic Reviews of Interventions was used to evaluate the quality of the retrieved RCTs. The following areas were covered by the Cochrane risk-of-bias assessment tool: sequence generation (selection bias), allocation sequence concealment (selection bias), blinding of participants and staff (performance bias), blinding of outcome assessment (detection bias), incomplete outcome data (attrition bias), selective outcome reporting (reporting bias), and other possible sources of bias.

### Data acquisition

To prevent bias, each publication was extracted separately by two randomized authors, and any disagreements were resolved by a third reviewer. The study design, participant country, participant age, description of cryotherapy, control groups, outcomes, and time points were among the characteristics extracted from the studies. Each process followed the suggested techniques in Higgins & Cochrane (2020). ^15^^,^^16^

### Data analysis

Because of the small number of included trials, a planned meta-analysis using a random effects model was not possible. Mean differences and 95% confidence intervals are shown. RevMan 5.4 software (Cochrane Collaboration) was used for all analyses. Arguments between the reviewers were handled by a third reviewer (MJJT). We planned subgroup analyses to examine the effects of different types and doses of cryotherapy, as well as the two forms of CRS (one with polyposis and one without). Furthermore, sensitivity analyses were performed to determine whether a significant risk of bias affected the estimates. We aimed to use meta-regression for subgroup and sensitivity analyses if feasible (i.e., at least 10 trials were analyzed); otherwise, qualitative analysis might have been performed in accordance with the guidelines. ^17^

### Publication bias

For <10 pooled studies, publication bias evaluation is unreliable according to Egger *et al.* As a result, we were unable to use Egger’s test for funnel plot asymmetry to evaluate publication bias in the current review. ^18^^,^^19^

## Results

### Search results

**Figure 1.**
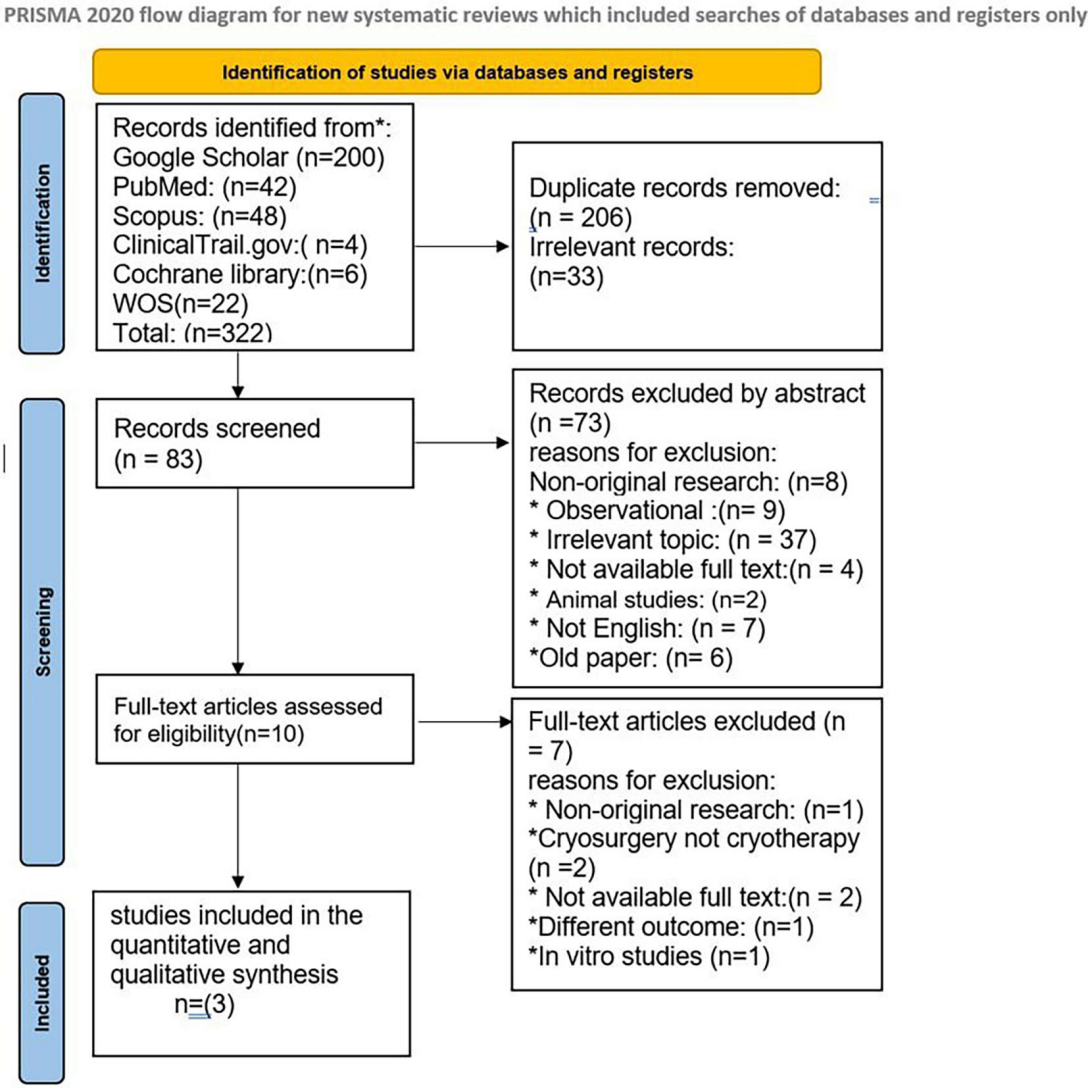
PRISMA flow chart for included and excluded studies.

**Figure 2.**
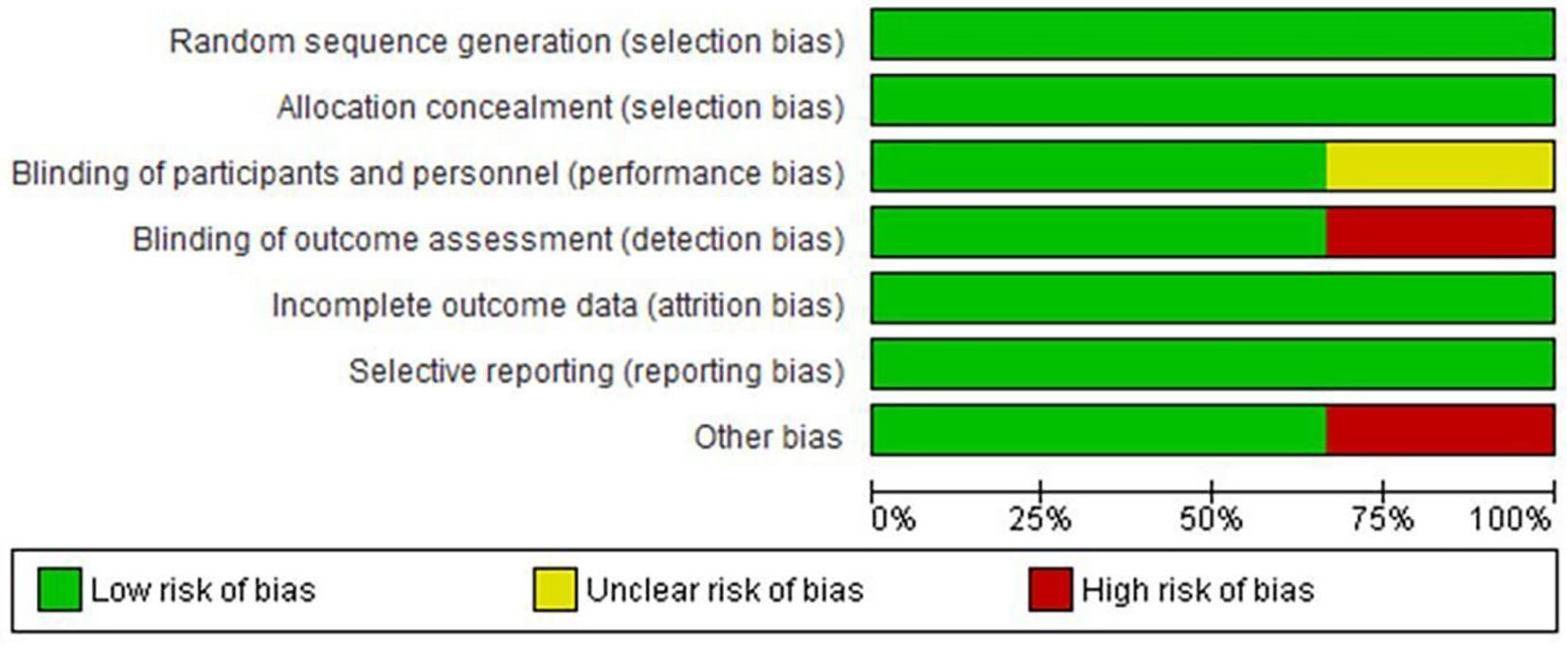
Risk of bias graph: review authors' judgements about each risk of bias item presented as percentages across all included studies.

**Figure 3.**
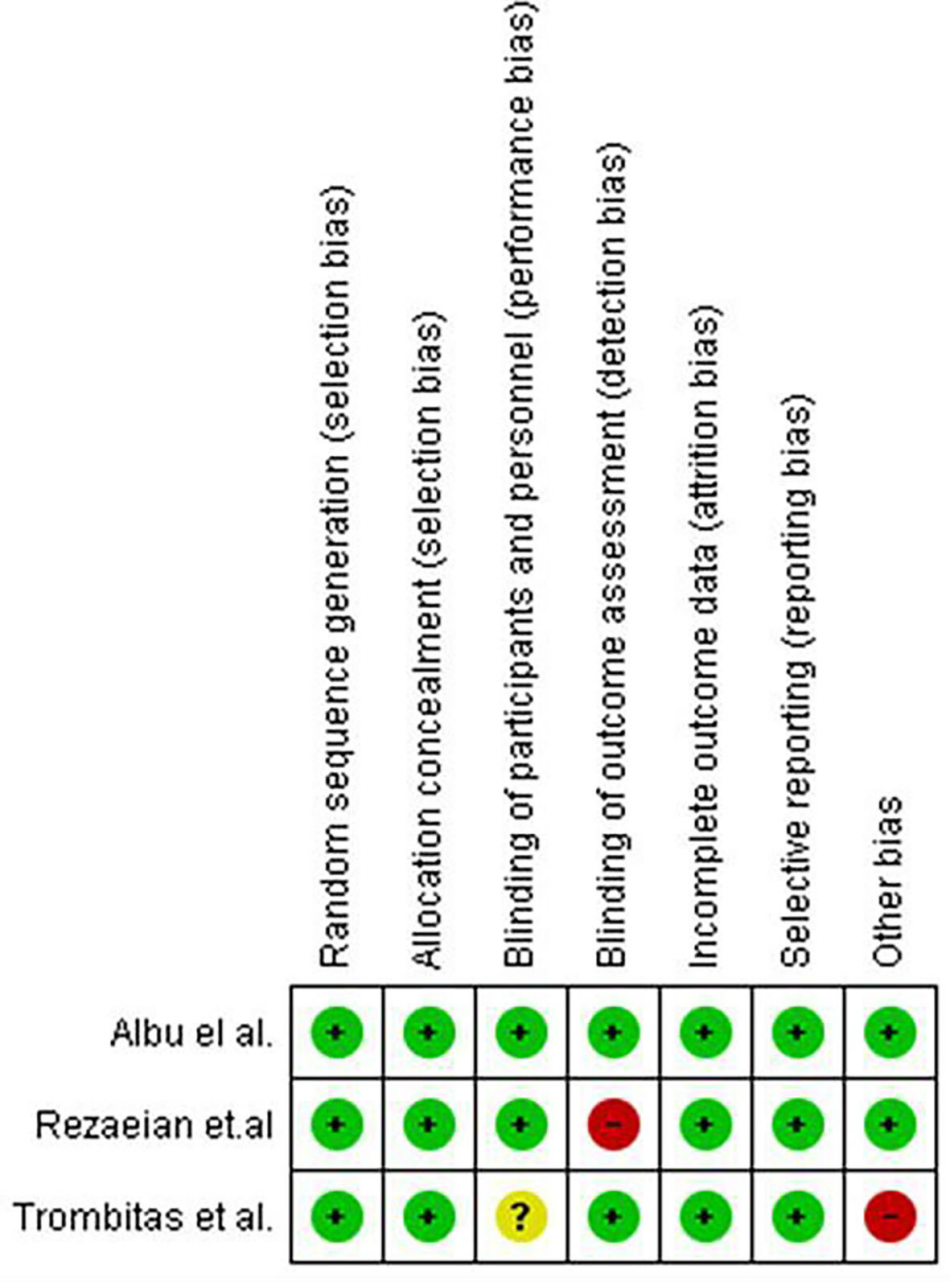
Risk of bias summary: review authors' judgements about each risk of bias item for each included study.

Our search yielded 322 results; 206 duplicates were eliminated, and the remaining 83 abstracts were reviewed. Three randomized controlled trials were incorporated after 10 prospective full texts were evaluated. Figure 1 in this review provides an illustration of the filtration procedures. According to the Cochrane risk-of-bias tool, all included studies were scored as low risk ( Figures 2 and 3).

### Characteristics of included studies

Three of the articles met the inclusion criteria. Table 1 summarizes the characteristics of the included studies. There were 85 chronic rhinosinusitis patients in the entire study population among all papers that were chosen, with approximately equal representation of men and women (54 males and 57 females). In all studies, the average age of the study population was 42.65±14 years.

**Table 1.** Characteristics of the included studies.

Study ID	Year	Groups	Sample Size	Age (Mean ±SD)	Sex (Male %)	Characteristics of patients	Pre-operative assessment of the patient	Follow up Duration	Conclusion
Rezaeian ^22^	2017	SCT	19	42.80 ±16.13	10 (50%)	Adult patients with bilateral nasal polyposis (CRSwNP).	Lund-McKay and SNOT-22 scores	Up to 36 weeks	SCT can consider a good management of NP after functional endoscopic surgery. In addition, there are no serious adverse effects reported from this technique.
		Saline	18	43.65 ±14.27	8 (40%)				
Albu *et al*. ^21^	2016	SCT	18 18	40.23 ±16.45 40.23 6 16.45	10 (45%) 10 (45%)	Adult patients with CRS.	POSE and Lund-Kennedy	Up to 12 weeks	SCT associated with significant reduction in synechia, edema of the mucosa, polypoid changes, and narrowing of the ostia in the whole follow-up period.
		Saline							
Trombitas *et al*. ^20^	2019	SCT	23 23	44.5 44.5	11 (42.3%) 11 (42.3%)	Patient with bilateral CRS without polyps (CRSsNP).	Lund-McKay score	Up to 12 months	SCT was associated with better outcome on MMA diameter, histological analysis, nasal obstruction and discharge with no any visual complications.
		Saline							

CRS, Chronic Rhinosinusitis, CRSsNP, Chronic Rhinosinusitis without nasal polyposis; CRSwNP, Chronic Rhinosinusitis with nasal polyposis; LM, Lund-Mackay; MMA, Middle Meatal Antrostomy; POSE, Perioperative Sinus Evaluation; SNOT-22, Sino-nasal Outcome Test-22.

### Operative techniques

Regarding the operative technique, devices, and post-operative management, the Brymill (Ellington, CT) CRY-AC-3 Cryogenic System was used in all included studies, and all patients received post-operative antibiotics as part of different post-operative protocols. The characteristics of the surgical and post-operative protocols are summarized in Table 2.

**Table 2.** Characteristics of the Surgical technique, cryotherapy used and post-operative management.

Article	Used SCT	Surgical technique and procedure	Post-operative management	
			Antibiotics	Others
Rezaeian ^22^	Brymill (Ellington, CT) CRY-AC-3 Cryogenic System	Uncinectomy, Infundibulectomy, Middle meatal antrostomy, Frontal sinusotomy, Sphenoidotomy, and Total ethmoidectomy.	Co-Amoxiclav 50 mg/kg TDS for 5 days.	Prednisolone 1 mg/kg/24 h for 5 days. Nasal steroids thrice daily for 1 month.
Albu *et al*. ^21^	Brymill (Ellington, CT) CRY-AC-3 Cryogenic System	Endoscopic sinus surgery (details not mentioned).	Oral broad-spectrum antibiotics for about 4 weeks.	Saline nasal irrigation for 4 weeks. Topical steroids for 8 weeks.
Trombitas *et al*. ^20^	Brymill (Ellington, CT) CRY-AC-3 Cryogenic System	Uncinectomy, Middle meatal antrostomy, Anterior ethmoidectomy, and Septoplasty.	Oral broad-spectrum antibiotics for 10 days.	Vaseline gauze. Daily saline nasal irrigation for 4 weeks.

### Effect of spray cryotherapy after endoscopic sinus surgery

Owing to the heterogeneity of the data regarding the measurements of post-operative improvement, conducting a meta-analysis was inappropriate. However, spray cryotherapy, according to Rezaeian (2018), was related to -16 and -77 reductions in Lund-McKay and SNOT-22 scores after 36 weeks of follow-up, in contrast to the placebo, which showed -10.4, -65. This outcome was also demonstrated by the POSE and Lund-Kennedy scores. Additionally, adhesions were more frequent on the control side than on the cryotherapy side. One middle meatus with a decreased dimension was reported in an SCT patient, compared to eight middle meatal antrostomies with stenoses in the control group at the final follow-up. In the Trombitas study, post-operative stenosis is defined as when the MMA diameter is less than 6 mm.

### Effect of SCT on post-operative nasal discharge, crustations and histological difference

According to Trombitaș *et al*., ^20^ the spray cryotherapy (SC) group significantly outperformed the control group in terms of nasal obstruction and discharge. Furthermore, the placebo side was linked to mononuclear cell infiltration, edema with collagen fiber dislocation, epithelial hyperplasia, goblet cells, and persistent squamous metaplasia on top of the epithelial hyperplasia, whereas the SC side was linked to superior collagen fiber organization. Similarly, according to Albu *et al*. ^21^ throughout the entire follow-up period, the side that had received cryotherapy showed considerably less edema of the mucosa, polypoid alterations, adhesions, and ostia narrowing. However, both treatments had identical distributions of discharge and crusting.

### Post-operative complications of spray cryotherapy

According to Albu *et al*., ^21^ there were no adverse effects (for example, hemorrhage or infection) in either group during the trial period, and this conclusion was also demonstrated by Rezaeian. ^22^ Furthermore, Trombitas *et al*. ^20^ investigated the visual assessments as SCT was sprayed around the orbit and found no pain, visual field loss, visual acuity, color perception, diplopia, ocular motility, visual acuity, globe displacement, or swelling.

## Discussion

The main goal of this study was to investigate the role and impact of SCT in chronic rhinosinusitis with or without nasal polyps following endoscopic sinus surgery. The most obvious finding to emerge from this study was that the overall effects of SCT are favorable, promising, and associated with good outcomes and better healing. SCT is a new post-operative modality that began with Dr. Albu and his team, ^21^ who used SCT following ESS based on a previous study conducted by Krimsky *et al*., ^23^ who used SCT to treat glottic and subglottic narrowing. However, Albu *et al*. ^21^ reported some limitations, such as a lack of subjective outcomes in the post-operative assessment, a short follow-up period, and the need to divide patients into two groups, with and without polyposis, to evaluate the overall impact of SCT on the healing of different diseases. Following the Albu study, Rezaeian ^22^ and Trombitas *et al*. ^20^ Three studies used the same SCT protocols. However, Rezaeian’s study had a 36-week follow-up period and only included patients with CRS with nasal polyposis. In addition, Trombitas’ study showed a longer follow-up period of 12 months (52 weeks), and it also measured mucosal histology pre- and post-operatively, which provided cellular evidence besides the clinical evidence regarding the healing outcome.

The present study investigated chronic rhinosinusitis with and without nasal polyposis. Despite differences in etiology and phenotype, many therapies for chronic rhinosinusitis are initiated in clinical practice without knowing a patient’s “polyp status.” Determining the type of CRS does not necessarily recommend therapy modifications. Lee-Yee *et al*. examined patients with and without polyps together in the first examination of treatment results, followed by a subgroup analysis, as we did. ^24^

The main symptoms of CRS are nasal blockage/obstruction/congestion and nasal discharge (anterior/posterior nasal drip) ^25^ and SCT shows a great reduction in all rhinosinusitis scores, lesser post-operative adhesions, discharge, side effects, inflammatory cell infiltration, and better collagen fiber arrangements in comparison with placebo, which could be explained by the fact that cryotherapy induces disruption of endothelial damage, thrombosis, and ischemia, and improves mucosal healing and decreases granulation tissue formation. ^26^ On the histological level, it resulted in better collagen organization and reduced keratinization, and long-term observation documented the absence of scarring and stricture formation. ^27^

Greenwald *et al*. ^28^ studied the safety and efficacy of endoscopic sinus surgery in the treatment of esophageal cancer and found no adverse effects, which is comparable to our findings. Using SCT in airway surgery, on the other hand, has been linked to problems such as barotrauma, pneumomediastinum, nitrogen gas embolism, and pneumothorax. ^29^ However, none of these side effects were reported in the trials included in the present study. As a result, SCT as a post-ESS treatment appears to be safe. However, further studies with longer follow-up periods are required to determine the safety of SCT.

One of the main post-operative complications of ESS in CRS is recurrence, which was not reported in any of the included trials for a 12-month follow-up period. However, in a study conducted on the recurrence of nasal polyposis, six-months after ESS recurrence was 35% and after 18-months it was 40%. ^30^ In other studies, the recurrence rate reached up to 60% after 18 months of follow-up, indicating that the percentage of recurrence increased over time; therefore, longer follow-up is needed to determine the recurrence ratio after SCT.

In accordance with the present results, a previous study conducted by Gorelik *et al*. demonstrated that both cryotherapy and radiofrequency had better outcomes than a placebo. ^29^ Moreover, SCT has been shown to improve post-operative outcomes in different ENT surgeries, including those for malignant airway disease, as demonstrated by a study conducted by Browning *et al*. ^31^ Browning *et al*. showed that SCT is safe and demonstrated better outcomes in post-operative respiratory complications from malignancies, such as dyspnea/hypoxia, granulation tissue, and bleeding tissue. Other advantages of using SCT in the management of airway diseases have been reported in many other studies. ^23^^,^^32^^,^^33^

One of the limitations of this study was the lack of data on the clinical history of the included patients, which could alter the patients’ results and outcomes. For example, diabetes can slow down the healing process. Furthermore, the Global Allergy and Asthma Network of Excellence epidemiological study found a strong link between asthma and CRS, ^34^ which raises two concerns: What was the prevalence of asthma in the included studies? How does cryotherapy affect the symptoms of asthma?

## Conclusions

The present systematic review was designed to determine the impact of SCT on ESS following chronic rhinitis; the results of this investigation show that SCT is associated with better healing and fewer negative complications such as synechia, edema, obstruction, crusting, and inflammation. Moreover, the findings of this study contribute to existing knowledge on the advantages of using cryotherapy in medicine in general, and specifically in post-operative airway surgery. Nevertheless, more research on this topic needs to be undertaken with osteitis, osteogenesis, and visual side effects, with a suitable assessment to reveal any adverse consequences associated with endoscopic SCT.

## Data Availability

All underlying data are available as part of the article and no additional source data are required. Figshare: PRISMA checklist for ‘The safety and efficacy of spray cryotherapy after endoscopic sinus surgery in chronic rhinosinusitis: A systematic review of randomized controlled trials’, https://www.doi.org/10.6084/m9.figshare.24661968. ^14^ Data are available under the terms of the Creative Commons Attribution 4.0 International license (CC-BY 4.0)
