# Supplementary material for: The safety and efficacy of spray cryotherapy after endoscopic sinus surgery in chronic rhinosinusitis: A systematic review of randomized controlled trials
Source: F1000Res. 2024 Jun 5;13:4. Originally published 2024 Jan 3. [Version 2] doi: 10.12688/f1000research.143321.2 (PMC11494270; doi:10.12688/f1000research.143321.2)
Supplement: Peer review history

# Reviewer report: Reviewer response for version 2

**DOI:** 10.5256/f1000research.167402.r330039

**Published:** 2024-10-21

**Authors:** Antonino Maniaci

**Affiliations:** 1 Department of Medicine and Surgery, University of Enna Kore, Research Committee of Young Otolaryngologists of the International Federation of Otorhinolaryngological Societies, Enna, Italy

Introduction:

 - improve the description of spray cryotherapy by expanding on what patients face during recovery and aftercare post-endoscopic sinus surgery.

 - Elaborate on spray cryotherapy – how it works, the theoretical mechanisms of action and previous uses in other fields cite doi:10.21037/jtd.2017.01.55.

 - Specify more precisely on what the review focus at end of introduction.

 Methods:

 - justify the decision to include only randomized controlled trials

 - This is of course a strength in the evidence, but this may have limited the number of studies that could be included.

 - Explanation of how data were extracted from the included studies and their synthesis

 - Think about prospectively registering review protocol ( e. in PROSPERO).

 Results:

 - explain why It was not possible to conduct a meta-analysis as there was considerable heterogeneity but one option would be provide more detailed qualitative synthesis of the results across studies.

 - Describe the risk of bias assessment in more detail for all included studies;

 Discussion:

 - Further discuss some of the possible mechanisms by which spray cryotherapy might lead to a better post-operative result.

 - Present implications for clinical practice and future directions of research in more depth. describe the useful application of subjective questionnaire to follow-up the response. cite doi:10.1007/s00405-023-07855-8

 - Further specify the limitations, especially regarding the low number of studies included and short periods for follow-up.

Are the rationale for, and objectives of, the Systematic Review clearly stated?

Yes

Is the statistical analysis and its interpretation appropriate?

Yes

If this is a Living Systematic Review, is the ‘living’ method appropriate and is the search schedule clearly defined and justified? (‘Living Systematic Review’ or a variation of this term should be included in the title.)

Yes

Are sufficient details of the methods and analysis provided to allow replication by others?

Yes

Are the conclusions drawn adequately supported by the results presented in the review?

Yes

Reviewer Expertise:

minor revisions

I confirm that I have read this submission and believe that I have an appropriate level of expertise to confirm that it is of an acceptable scientific standard, however I have significant reservations, as outlined above.

# Reviewer report: Reviewer response for version 1

**DOI:** 10.5256/f1000research.156967.r275424

**Published:** 2024-05-29

**Authors:** Serafín Sánchez-Gómez

**Affiliations:** 1 Department of Otorhinolaryngology and Head and Neck Surgery, Virgen Macarena University Hospital, Seville, Spain

The authors present a systematic review study that evaluated the effects of spray cryotherapy (SCT) following endoscopic surgery for chronic rhinosinusitis. Three randomized clinical trials including a total of 85 patients were selected.

 Several decades after the introduction of endonasal endoscopic techniques for the treatment of CRS, adverse events and complications in the surgical field continue to occur despite technical and instrumental improvements. Therefore, it is pertinent to investigate new ways to reduce these events and complications that deteriorate the quality of life of patients, requiring meticulous postoperative care and sometimes leading to reoperations.

 The authors present a study that compiles the experience of using spray cryotherapy on the surgical bed after endonasal endoscopic surgery, selecting three clinical trials that met the inclusion and exclusion criteria. The authors applied an extremely rigorous selection criterion for articles, including only those containing randomized and blinded clinical trials. This design is not well-suited for surgical procedures and introduces significant uncertainties about the reliability of the data included in the clinical trials. The inclusion of other well-designed prospective studies would have been acceptable.

 The methodology of the systematic review is pertinent and correct. It would have been desirable for the authors to submit their project to be included in the PROSPERO database of systematic reviews. Nevertheless, the use of the PRISMA statement and Cochrane methodology ensures that the authors have used the best methods to adequately conduct a systematic literature review. The presentation of results in the PRISMA flowchart and tables is appropriate and sufficient.

 Given the novelty of spray cryotherapy in the field of ESS, the authors could have provided more information about this therapy in the Introduction, as adverse events and complications are well-known.

 The results of their systematic review showed that SCT was associated with better outcomes in Lund-McKay, SNOT-22, POSE, and Lund-Kennedy scales compared to placebo. It was also associated with less edema, obstruction, crusting, and inflammation. No adverse effects of SCT were reported. These are perfectly suitable outcome variables for the review's objective.

 The authors have identified the main five weaknesses of the study:

 1. The sample size was small (only 3 studies with a total of 85 patients). Larger studies are needed.

 2. Follow-up periods were relatively short, up to a maximum of 12 months. Longer follow-up studies are needed.

 3. No information was provided on the clinical history of the included patients, which could have affected the results.

 4. Potential long-term side effects of SCT, such as osteitis or other ocular complications, were not evaluated.

 5. Only English articles were included, which could have introduced language bias. Studies in other languages should also be considered.

 Throughout the draft, a series of errors have been detected that should be resolved:

 a.           In the Abstract, a consideration needs improvement: The phrase "Endoscopic sinus surgery (ESS) is the preferred treatment because of its few adverse effects" significantly limits the objective of the surgery if it is not stated that the reason for choosing this technique is due to achieving the best results, not only due to adverse effects.

 b.           In the 6th line of the Introduction, in the phrase "...with validation by telephone or interview.", there is an unnecessary period at the end of the phrase.

 c.           The third inclusion criterion in the Methodology section has "Only in English" capitalized, whereas the first criterion is in lowercase. The decision on capitalization should be consistent.

 d.           In the first paragraph of the Discussion, the phrase is incomplete or has punctuation errors: "Following the Albu study, Rezaeian22 and Trombitas et al.20 Three studies..."

 e.           The phrase in the Conclusion column of Table 1 contains English writing errors: "SCT was associated with better outcomes in MMA diameter, histological analysis, nasal obstruction, and discharge, with no visual complications."

 f.            The phrase in the paragraph starting with Greenwald et al. should be corrected as the surgical technique does not correspond to the disease.

 g.            In the phrase "Other advantages of using SCT in the management of airway diseases have been reported in many other studies," the word "many" is excessively emphatic and should be omitted.

 h.           The first sentence of the Conclusions, "The present systematic review..." could be better expressed as "The present systematic review was designed to determine the impact of SCT on ESS in patients with chronic rhinosinusitis" or “...following endoscopic surgery for chronic rhinosinusitis”

Are the rationale for, and objectives of, the Systematic Review clearly stated?

Yes

Is the statistical analysis and its interpretation appropriate?

Not applicable

If this is a Living Systematic Review, is the ‘living’ method appropriate and is the search schedule clearly defined and justified? (‘Living Systematic Review’ or a variation of this term should be included in the title.)

Not applicable

Are sufficient details of the methods and analysis provided to allow replication by others?

Yes

Are the conclusions drawn adequately supported by the results presented in the review?

Yes

Reviewer Expertise:

Rhinology, cochlear implants

I confirm that I have read this submission and believe that I have an appropriate level of expertise to confirm that it is of an acceptable scientific standard, however I have significant reservations, as outlined above.

# Response

**Published:** 2024-06-01

**Authors:** Abdulqadir Nashwan

**Affiliations:** Hamad Medical Corporation, Qatar

Comment (Lines 90-95): At the same time, general information about chronic rhinosinusitis seems somewhat redundant. It may be worth adding information about the difficulties and challenges of postoperative care of the nasal mucosa after ESS, postoperative access to prevent scarring and synechiae formation.

 Response: We appreciate the suggestion to provide more details on postoperative care. We will add information focusing on these challenges and how they can be managed effectively, as highlighted in references 5 and 6.

 Comment (Resolved Line 239): The paragraph beginning "Greenwald et al." is most likely a misspell: replace endoscopic sinus surgery with spray cryotherapy.

 Given the researchers' stated goal, it seems rational to replace chronic rhinitis with chronic rhinosinusitis in the conclusion section.

 Response: The corrections have been made to accurately reflect the treatment discussed and the condition being addressed in the conclusion.

 Comment: The authors applied an extremely rigorous selection criterion for articles, including only those containing randomized and blinded clinical trials. This design is not well-suited for surgical procedures and introduces significant uncertainties about the reliability of the data included in the clinical trials. The inclusion of other well-designed prospective studies would have been acceptable.

 Response: We use randomized control trials as our inclusion criterion to ensure the highest quality data. Despite an extensive search for additional relevant studies, none met our rigorous standards, not even non-randomized clinical studies.

 Comment (Lines 99-101): Given the novelty of spray cryotherapy in the field of ESS, the authors could have provided more information about this therapy in the Introduction, as adverse events and complications are well-known.

 Response: We acknowledge this oversight and will revise the Introduction to include more comprehensive details about spray cryotherapy, especially regarding its adverse events and complications.

 Comment (Line 36): In the Abstract, a consideration needs improvement: The phrase "Endoscopic sinus surgery (ESS) is the preferred treatment because of its few adverse effects" significantly limits the objective of the surgery if it is not stated that the reason for choosing this technique is due to achieving the best results, not only due to adverse effects.

 Response: We will adjust the wording to clarify that the choice of ESS is based on its effectiveness in achieving the best outcomes, not merely its few adverse effects.

 Comment (Line 121): The third inclusion criterion in the Methodology section has "Only in English" capitalized, whereas the first criterion is in lowercase. The decision on capitalization should be consistent.

 Response: The capitalization will be corrected to maintain consistency throughout the document.

 Comment (Line 223): In the first paragraph of the Discussion, the phrase is incomplete or has punctuation errors: "Following the Albu study, Rezaeian22 and Trombitas et al.20 Three studies..."

 Response: This error will be rectified to ensure the sentence is complete and clear.

 Comment (Line 261): In the phrase "Other advantages of using SCT in the management of airway diseases have been reported in many other studies," the word "many" is excessively emphatic and should be omitted.

 Response: The word "many" will be removed to avoid undue emphasis and maintain a neutral tone.

 Comment (Lines 270-271): The first sentence of the Conclusions, "The present systematic review..." could be better expressed as "The present systematic review was designed to determine the impact of SCT on ESS in patients with chronic rhinosinusitis" or “...following endoscopic surgery for chronic rhinosinusitis”

 Response: We will revise the conclusion to reflect these suggestions for greater clarity and precision in presenting the study’s objectives and outcomes.

# Reviewer report: Reviewer response for version 1

**DOI:** 10.5256/f1000research.156967.r261156

**Published:** 2024-04-18

**Authors:** Sergei Karpischenko, Ekaterina Savchenko

**Affiliations:** 1 Pavlov First Saint Petersburg State Medical University, Saint Petersburg, Russian Federation

Mohammad J. J. Taha et al. present an important and interesting systematic review to analyse data from randomised clinical trials on the outcomes of spray cryotherapy (SCT) after endoscopic sinus surgery (ESS) in patients with chronic rhinosinusitis with and without polyps.

 Functional endoscopic surgery of the nasal cavity and paranasal sinuses is currently the leading method of surgical treatment of chronic rhinosinusitis. In the postoperative period it is necessary to correct inflammatory and regenerative processes to reduce postoperative reactive phenomena, prompt healing, reduce the risk of complications. The accumulated clinical and scientific data show that despite the diversified preventive measures after ESS local complications in the nasal cavity remain an urgent problem.

 Spray cryotherapy is a possible treatment method that can reduce complications after ESS.

 Abstract

 Covers the aims, methodology, overall results and conclusions.

 Introduction

 Aspects of endoscopic sinus surgery and the relevance of adjunctive postoperative care are covered in detail and convincingly. The authors provide information on the place of the problem in otorhinolaryngology and the health care system, and recall the impact of chronic rhinosinusitis on quality of life.  At the same time, general information about chronic rhinosinusitis seems somewhat redundant. It may be worth adding information about the difficulties and challenges of postoperative care of the nasal mucosa after ESS, postoperative access to prevent scarring and synechiae formation. It is probably worth presenting information about the existing possible solutions to these problems, emphasising that this issue has no unambiguous solution.

 Methodology

 A systematic review of randomised controlled trials was conducted according to the PRISMA checklist. The filtering stages of the studies were carried out according to Cochrane criteria. Which indicates the compliance of the publication with international standards. The inclusion criteria are relevant to the research objectives. The process of material collection is described in an accessible and consistent manner and provides insight into the sample obtained. Each publication was extracted separately by two independent authors and any disagreements were resolved by a third reviewer. The size of the sample obtained influenced the planned statistical processing methods, which did not affect the quality of the article or its informative value in any way.

 Results

 As a first figure, a PRISMA flow chart of included and excluded studies is presented, which allows to assess the amount of work undertaken in the process of selecting studies for the systematic review.

 Subsequently, the risks of bias for each included study are given graphically.

 A table of studies provides a comprehensive overview of the patient groups, participant characteristics, outcomes and time periods of follow-up.

 The effects of cryotherapy administered after endoscopic sinus surgery are systematically analysed based on the SNOT-22, Lund-McKay, POSE and Lund-Kennedy scoring scales.

 Discussion

 An extended review of the literature on the above topic was performed and references to similar studies are provided.

 The paragraph beginning "Greenwald et al." is most likely a misspell: replace endoscopic sinus surgery with spray cryotherapy.

 Given the researchers' stated goal, it seems rational to replace chronic rhinitis with chronic rhinosinusitis in the conclusion section.

 In summary, this is a systematic review that is an important addition to the literature. As the authors correctly note, cryotherapy after endoscopic sinus surgery is a promising method that requires follow-up RCTs.

Are the rationale for, and objectives of, the Systematic Review clearly stated?

Yes

Is the statistical analysis and its interpretation appropriate?

Yes

Are sufficient details of the methods and analysis provided to allow replication by others?

Yes

Are the conclusions drawn adequately supported by the results presented in the review?

Yes

Reviewer Expertise:

Clinical and scientific interests: endoscopic sinus sugery, laser surgery in otorhinolaryngology, laryngeal and tracheal microsurgery, otosurgery, oncology, dacryology.

We confirm that we have read this submission and believe that we have an appropriate level of expertise to confirm that it is of an acceptable scientific standard.
